# Using audit and feedback to increase clinician adherence to clinical practice guidelines in brain injury rehabilitation: A before and after study

**DOI:** 10.1371/journal.pone.0213525

**Published:** 2019-03-13

**Authors:** Laura Jolliffe, Jacqui Morarty, Tammy Hoffmann, Maria Crotty, Peter Hunter, Ian. D. Cameron, Xia Li, Natasha A. Lannin

**Affiliations:** 1 College of Science, Health Engineering, La Trobe University, Bundoora, Victoria, Australia; 2 Alfred Health, Caulfield, Victoria, Australia; 3 Centre for Research in Evidence-Based Practice, Bond University, Robina, Queensland, Australia; 4 Flinders University, Bedford Park, Adelaide, Australia; 5 John Walsh Centre for Rehabilitation Research, Kolling Institute, University of Sydney, Camperdown, Sydney, Australia; 6 Department of Mathematics and Statistics, La Trobe University, Bundoora, Victoria, Australia; University of Montreal, CANADA

## Abstract

**Objective:**

This study evaluated whether frequent (fortnightly) audit and feedback cycles over a sustained period of time (>12 months) increased clinician adherence to recommended guidelines in acquired brain injury rehabilitation.

**Design:**

A before and after study design.

**Setting:**

A metropolitan inpatient brain injury rehabilitation unit.

**Participants:**

Clinicians; medical, nursing and allied health staff.

**Interventions:**

Fortnightly cycles of audit and feedback for 14 months. Each fortnight, medical file and observational audits were completed against 114 clinical indicators.

**Main outcome measure:**

Adherence to guideline indicators before and after intervention, calculated by proportions, Mann-Whitney U and Chi square analysis.

**Results:**

Clinical and statistical significant improvements in median clinical indicator adherence were found immediately following the audit and feedback program from 38.8% (95% CI 34.3 to 44.4) to 83.6% (95% CI 81.8 to 88.5). Three months after cessation of the intervention, median adherence had decreased from 82.3% to 76.6% (95% CI 72.7 to 83.3, *p*<0.01). Findings suggest that there are individual indicators which are more amenable to change using an audit and feedback program.

**Conclusion:**

A fortnightly audit and feedback program increased clinicians’ adherence to guideline recommendations in an inpatient acquired brain injury rehabilitation setting. We propose future studies build on the evidence-based method used in the present study to determine effectiveness and develop an implementation toolkit for scale-up.

## Introduction

Acquired brain injury is a leading cause of disability in adults [1] with a large proportion of patients requiring rehabilitation [2]. Consistent with other areas of health care, neurological rehabilitation has been observed to vary in quality between services [3, 4]. Clinical practice guidelines provide recommendations to assist clinicians make evidence-informed decisions about the interventions they provide [5–7]. Despite the availability of such guidelines, auditing suggests that rehabilitation clinicians do not routinely provide care consistent with guideline recommendations [8]. Audit and feedback has been recommended as an intervention capable of increasing the uptake of evidenced-based recommendations by clinicians [9–11].

A growing number of researchers are trialing audit and feedback interventions to promote the use of evidence in rehabilitation, however outcomes for improving clinician adherence has been mixed. The use of implementation interventions in rehabilitation is undoubtedly a positive step forward, nevertheless, critical reflection on the effectiveness of different interventions is key. Specific to audit and feedback interventions, two systematic reviews have synthesised the evidence on effectiveness; these reviews suggest limited to modest improvements occur at best [12,13]. The latest Cochrane systematic review concluded that audit and feedback generally produces small, but potentially important improvements [12]. This is consistent with a second meta-analysis, which found modest improvements on quality outcomes [13]. These reviews [12, 13] suggest the need for clear definitions of goal-behaviors, and triangulation of data collection to improve the effect of audit and feedback interventions. They also suggested that the characteristics of the feedback component of future studies should be identified so as to build an understanding of the causal mechanisms underpinning audit and feedback as an intervention [12–14].

Prior audit and feedback interventions to increase adherence to guidelines in rehabilitation have been provided infrequently or at low ‘dose’. For example, to improve the implementation of transport training after stroke, McCluskey and colleagues [15] delivered a single audit and feedback cycle in their knowledge translation program, while Kristensen & Hounsgaard [16] provided four cycles over 15 months, and Vratsistas-Curto et al [17] provided four cycles over 4 years. What remains unknown is the effect of audit and feedback when it is provided at a higher dose (such as weekly or fortnightly). A further limitation of the rehabilitation studies to date is that none triangulated their audit information; triangulation occurs by gathering information from multiple sources and while missing from the rehabilitation.

Studies outside of rehabilitation also suggest that it is important to strategically plan the method of feedback delivery; for example, nurses reported feeling ‘exasperated’ and ‘angry’ when they received feedback they perceived as critical [18]. Few studies have reported the use of a theoretical underpinning to their feedback delivery [12, 13, 19]. In contrast, LaVigna and colleagues [20] deliberately adopted a ‘non-aversive approach’ when working with staff in quality improvement cycles, and developed a form of audit and feedback known as periodic service review [20, 21]. Periodic service review has its base in both total quality management [22] and organizational behavior management [23, 24], and differs from other auditing approaches used in prior rehabilitation studies, since it is undertaken at a high dose, uses positive support strategies during feedback, and actively involves staff in the process [21]. It remains unknown if this approach to audit and feedback would increase adherence to guidelines in rehabilitation, where prior audit and feedback studies have not.

Therefore, the aim of this study was to evaluate the impact of a prospective audit and feedback program on adherence to acquired brain injury rehabilitation guidelines. We sought to understand whether:

1. frequent audit and feedback cycles (with positive behavioral support) increased clinician adherence to clinical practice guidelines in acquired brain injury
2. increases in adherence are maintained after the cessation of audit and feedback program
3. changes in adherence differ according to individual guideline indicators

## Method

### Design

**Fig 1:**
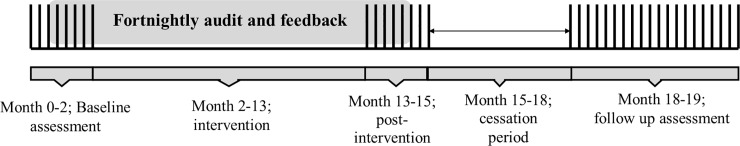
Design and flow of the study.

A before and after design with a 3-month follow-up was used to test the effect of a 14-month audit-feedback program in an inpatient rehabilitation setting. There were 8 assessments at baseline, 8 assessments at end of intervention and 20 assessments at follow-up. The study design and flow is depicted in (Fig 1). The administrative organization’s Human Research Ethics Committee approved this study prior to its commencement (Alfred Health Human Research Ethics Committee 355/14); a waiver of consent for participation was approved, meaning that all inpatients and all staff were involved for the duration of the study period.

### Settings and participants

This study was conducted between September 2014 and March 2016 in a newly established 42-bed acquired brain injury rehabilitation unit in metropolitan Melbourne, Australia. All clinicians (inclusive of nursing, medical, and allied health staff) working on the unit were included in this study and expected to attend each fortnightly feedback session as part of their usual workplace meeting commitments with support of management. Staffing ratios within the unit are presented in Table 1. At the time of this study, other passive knowledge translation interventions (including the availability of guidelines on each ward, and posters of best practice summaries) were also provided to clinicians.

**Table 1:** Staffing profile during intervention period.

Discipline	Average staffing ratio per 10 beds	Mean occasions of service per month per 10 beds
Allied Health Assistants	1.31	380
Clinical Psychology	0.33	61
Neuropsychology	0.53	70
Occupational Therapy	1.38	259
Nutrition	0.43	42
Prosthetics and Orthotics	0.14	34
Podiatry	0.05	5
Physiotherapy	1.46	237
Speech Pathology	0.86	175
Social work	1.01	131
Nursing	9.5	-
Specialist Rehabilitation Physician	0.625	-
Junior Medical Staff	1	-

### Intervention

A 14-month audit and feedback program was developed. Audit criteria were developed by two authors (NL, LJ) *a priori* from recommendations with high-quality (Grading of Recommendations Assessment, Development and Evaluation (GRADE) level one) evidence cited in stroke and traumatic brain injury clinical practice guidelines [25, 26] as well as the organization’s model of care and practice standards [27]. The resultant 114 observable criteria were mapped to 16 overarching guideline indicator areas for ease of communication with staff regarding performance. These guideline indicator areas included: behavioral support plans, care plans, continuity of care, discharge planning, equipment use, family education, goal setting, medical issues management, medical records, minimally conscious care, patient safety, personal care regimes, post traumatic amnesia management, roles and responsibilities, therapy interventions, and ward rounds. The organization set the target for staff to adhere to a minimum of 75% of applicable guideline indicators per patient prior to commencing the study.

**Fig 2:**
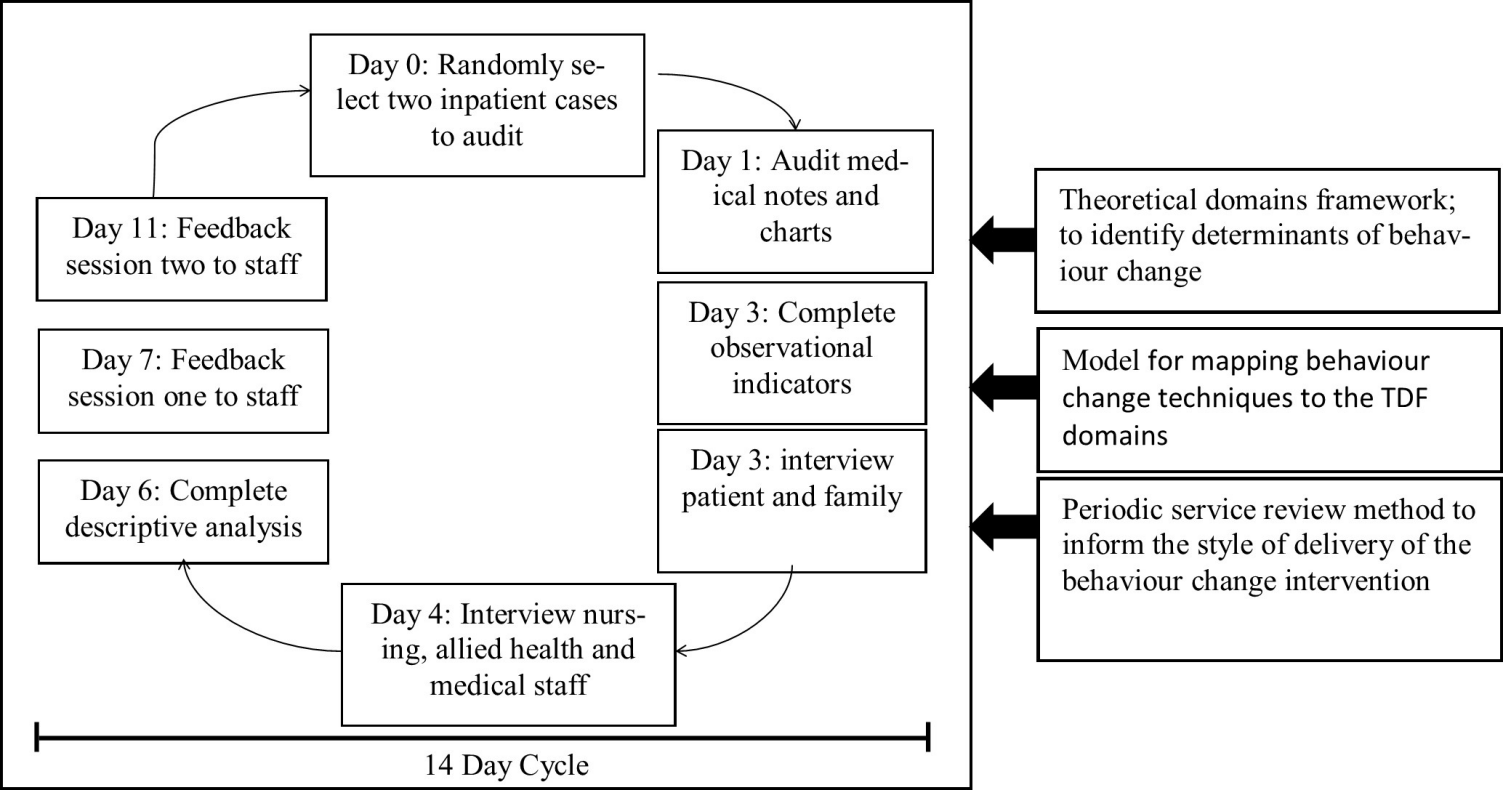
Flow of fortnightly intervention.

Our audit and feedback program was based on the periodic service review method developed by LeVigna et al[20]. By acknowledging that the clinical team are key to delivery of evidence-based rehabilitation, we aimed to improve and then maintain the quality of the service using positive behavioral approaches to staff management [21]. We adopted a non-aversive approach to working with the staff during the feedback session, making the clinicians the leaders of the change solutions [21, 23, 24]. The audit-feedback cycles were regular and frequent throughout the study period. Each fortnight, a research assistant randomly selected two patients on the rehabilitation unit (one from each of the two medical teams) and completed a) medical file audit; b) on ward observations; c) clinical staff interviews of three disciplines (allied health, nursing and medical); d) patient interview; and e) family / friend interviews. At the completion of both audits, descriptive statistics (proportion of criteria adherence) were calculated and prepared for the clinician feedback meeting. Feedback sessions were offered twice within each fortnight period to enable shift-working staff to attend. These 15-minute sessions provided the audit results to clinicians, and were delivered by the senior author (NL) an accepted member of staff. Following the feedback sessions, data were made available to all staff via a shared drive on the organization’s computer network. These audit-feedback cycles were repeated every two weeks for 14 months. The intervention is summarized in Table 2; please refer to (Fig 2) for the flow of the fortnightly intervention and (S1 Table) for the Standards for Reporting Implementation Studies.

**Table 2:** Intervention summary based on TIDieR, delivered by researchers.

Intervention components	Rationale	Mode of Delivery	Delivered to	When/how often
Evidence introductory education session, including target setting of 75% adherence	To familiarise staff with the audit/feedback intervention and increase awareness of guideline indicators	Face-to-face (group)	Doctors, nurses, allied health staff, patient support staff, reception staff	Each staff member attended one session, and once at each new staff induction to the ward
Point of care access to clinical practice guideline evidence	To educate staff about the guidelines and ensure access to the evidence underpinning guideline indicators	Documents loaded onto an e-reader device	Doctors, nurses, allied health staff, patient support staff	Ongoing
Educational summary of guideline indicators	To provide education about single guideline indicators and promote self-monitoring	Small summarised poster mailed participants, and poster documents placed on wall	Doctors, nurses, allied health staff, patient support staff, reception staff	Small summarised poster mailed fortnightly to all staff; A3 summarised poster placed on wall ongoing
Audit and group feedback	To focus staff on targets and progress, group discussion aided in process of care changes to increase adherence rates	Feedback presentation displayed rates graphically, feedback delivered face-to-face (group)	All available staff on shift at time of feedback presentation	Fortnightly auditing of cases, feedback delivered bi-weekly
Feedback to staff outside of scheduled feedback sessions	To update staff on progress and targets	Feedback provided one-on-one or email copy of feedback presentation. Fortnightly feedback was made available on the organisation’s share drive.	Staff who missed all the biweekly feedback sessions and requested an update	Adhoc, ~1 staff per fortnight

Audit data were triangulated, involving a medical file audit, interviews with clinical staff, and interviews with the patient and/or family. An example of an interview question with a clinical staff member is *“Can you identify the patient’s primary rehabilitation goals consistent with the documented goals from the interdisciplinary family meeting”*. If the clinician responded correctly, this item was deemed met and scored “yes” on the audit form. An example of a medical file audit indicator was *Does the patient receive 4*.*5–5 hours of therapy daily*? To score ‘yes’ for this item, on ward observations as well as review of the patient’s therapy timetable was completed. An example of an interview question with the patient and or family member is *“Did someone provide you with a tour of the unit when you first arrived on the ward”* The responses to these interviews (yes or no) were recorded on the audit form. (The data dictionary of audit criteria is available from author on request).

A cessation period of three months then ensued, in which no auditing or feedback occurred. In March 2016, n = 20 randomly selected inpatient cases were audited (consistent with the main audit method) to investigate guideline adherence following intervention cessation.

### Organizational context

The intervention was tailored to the organization, and designed to be multifaceted (to increase the likelihood of uptake) and frequent (to lower the fidelity gap). The core of the intervention (i.e. audit and feedback) was held consistent throughout the study (no adaptations); instead, the passive knowledge translation interventions (in particular, the education components) were tailored to address highlighted fidelity gaps each fortnight. For example, if auditing revealed low adherence to a guideline indicator, an evidence summary was created to increase staff awareness of the expected behavior. To understand the intervention *dose delivered* and *dose received*, we collected data on both number of staff employed (who would have received all passive knowledge translation components) and number of staff who attended the feedback sessions (referring to exposure to and uptake of the core intervention).

Our implementation intervention targeted behavior changes within both the individual (i.e., staff) and the organization. While the feedback was provided to staff, behavior change discussions held within feedback sessions took into consideration the context of the organization, the patient / family dyads and the national healthcare system). With staff leading the behavior changes, they held in-depth knowledge of the processes that controlled adoption of the guidelines within their organization, maximizing effect[28]. Our implementation targets were individual clinicians who worked within the rehabilitation unit, however, buy-in and support from management was an obvious factor impacting on implementation effectiveness. The Director of Rehabilitation, Director of Nursing Services and the Service Manager were asked to communicate support for guideline implementation to staff during orientation, at staff meetings, and via email throughout the intervention period.

### Outcome measures

The primary outcome was adherence to guideline indicators as measured by the audits. Consistent with the auditing which formed part of the intervention, this included triangulation of data from the medical file audits, unit based observations, and patient, staff, family interviews.

### Data analysis

Each fortnight, dichotomous data were recorded in an excel spreadsheet, and later imported into SPSS V24 for analysis. The mean adherence from audit data of month 0–2 was calculated to represent ‘baseline’ adherence. Mean adherence audit data from month 13–15 was calculated to represent ‘end of intervention’ adherence comparisons. Following intervention cessation (months 15–18), 20 randomly selected cases were audited (month 18–19) to calculate average (mean) adherence to assess if adherence was maintained or reduced. Where an audit item was not applicable to the selected case (i.e., if the selected case was not minimally conscious and therefore the minimally conscious care item(s) were not applicable), this item(s) was removed from the analysis for that period.

Median (95% confidence intervals) and Mann-Whitney U analyses were used to describe comparisons across all data due to the small sample size at each timepoint (n = 8, n = 8, n = 20 respectively) producing non-normally distributed data. Confidence intervals were calculated to highlight statistical significance where it existed, along with measures of variance around median differences (IQR). Chi square analysis for individual guideline indicator items were conducted to compare adherence across comparison points (given data was binary) with Fischer exact test statistic additionally reported due to small sample size[29]. To describe the data, mean (95% confidence intervals) and difference between means (95% confidence intervals) were also calculated and are presented in (S2 Table). The Bonferroni correction was applied to adjust the alpha level for all tests since multiple comparisons were made (with tests run for 230 comparisons, the alpha level was lowered to 0.0002). Refer to (Fig 2) for diagrammatic representation of analysis points.

Following quantitative analysis, narrative synthesis was undertaken to synthesise findings from our study with recommendations relating to conducting audit and feedback projects drawn from previously conducted systematic reviews [12,13]. Two authors [NL, LJ] extracted contributing factors which led to the success of the audit and feedback program into categories highlighted by these previous systematic reviews. All authors then reviewed and refined the list of factors.

## Results

During the study period, 58 clinical staff were employed with strong representation at fortnightly feedback sessions, mean of 67% (SD 8) attendance. Clinical profiles of patients audited at time point is presented in Table 3.

**Table 3:** Patient demographic characteristics of randomly selected patients included at each audit time point.

Characteristic	Time points		
	0–2 months *(n = 8)*	13–15 months; post intervention *(n = 8)*	18–19 months; follow-up *(n = 20)*
Diagnosis			
TBI, n (%)	3 (38)	4 (50)	7 (35)
Stroke, n (%)	4 (50)	3 (28)	7 (35)
Other*, n (%)	1 (12)	1 (12)	6 (30)
Gender			
Male, n (%)	6 (75)	6 (75)	16 (80)
Age, *mean years (sd)*	42 (16)	38 (17)	47 (15)
Length of stay *mean days*, *(min—max)*	193 (23–423)	106 (13–452)	147 (37–362)
Total FIM score at Admission (possible scores18-126), *median (IQR)*	27 (18.5, 42.5)	28 (20, 50.5)	33 (19,70.5)
FIM Cognitive Score at Admission (possible scores 5–35), *median (IQR)*	7.5 (5.5, 16.5)	8.5 (5, 16)	10 (5, 16)
FIM Motor Score at Admission (possible scores 13–91), *median (IQR)*	17.5 (13, 25)	18 (13.5, 37.5)	16 (61,13)

TBI = Traumatic Brain Injury

*Tumour and/or hypoxic brain injury.

The sustained audit and feedback program significantly increased clinician’s adherence to guideline recommendation from median 38.8% (95% CI 34.3 to 44.4) at baseline to 83.6% (95% CI 81.8 to 88.5) at the end of the intervention. Table 4 shows median total adherence at each time point. Following cessation of the audit and feedback program, clinician adherence levels decreased by 7% (95% CI .51 to 14.0) from the end of the intervention to follow up, however adherence to guideline indicators was maintained above the organization’s goal of 75% adherence.

**Table 4:** Median (IQR) of clinical practice guideline indicator adherence across measurement points, median differences between timepoints (95% Confidence Interval) and significance of the between group difference.

Adherence	Percent (%) of clinical practice adherence obtained at three time points (IQR)			Difference between groups; Mann-Whitney U, p-value*	
	0–2 months (baseline)	13–15 months (post intervention)	18–19 months (follow-up)	13–15 months minus 0–2 months	18–19 months minus 13–15 months
Total adherence (%)	38.8 (32.8, 65.1)	83.6 (78.4, 89.4)	76.6 (60.4, 88.6)	45.2 (95% CI 38.5 to 50.3) .000, *p =* 0.0001*	-7.0 (95% CI -0.5 to -14.0) 125, *p* = 0.0102

CPG = clinical practice guideline, CI = Confidence Interval

* statistically significant at the Bonferroni adjusted p-value 0.000217

Adherence differed across guideline indicators, with some indicators more susceptible to change with the audit and feedback program, and others that were not. For example, indicators related to ‘goal setting’, ‘therapy’ and ‘roles and responsibilities’ increased significantly during the intervention period, but this increase was not sustained at follow up. Conversely, adherence to most of the ‘ward round’ indicators did not improve during the intervention period. Refer to Table 5 (and S2 Table) for full indicator change results.

**Table 5:** Adherence to audited indictors (n = 114) at three audit time points and difference (Chi square) between time points.

Explicit audit indicators linked to model of care and/or clinical practice guideline recommendations	Adherence to audit criteria			Differences in adherence measured between time points			
	0–2 months (n = 8)	13–15 months; post intervention (n = 8)	18–19 months; follow-up (n = 20)	13–15 months minus 0–2 months		18–19 months minus 13–15 months	
	n	n	N	*p* value (Fischer exact statistic)	Cramer’s V	*p* value (Fischer exact statistic)	Cramer’s V
**Behavioural support plan**							
1: Patient behavioural support plan is known to the family and informal carers [Model of care recommendation]	3	1	5	*	*	1.0	.289
2: An admission screen of behavioural support requirements has taken place [26]	3	8	19	.026	.674^‡^	1.0	.122
3: Patient behavioural support plan is in place [26]	2	3	12	.196	.600^‡^	*	*
4: The implementation of strategies documented in the patient behavioural support plan occurs [26]	2	3	12	.429	.548^‡^	*	*
5: Patient behavioural support plan is known to staff [26]	7	8	18	*	*	*	*
6: Antecedent behaviours are known to staff [26]	2	1	10	1.0	.333^†^	.154	.452^†^
**Care plan**							
1: Family are able to identify primary rehabilitation goals consistent with documented goals from interdisciplinary family meeting [Model of care recommendation]	3	4	8	.444	.478^†^	.516	.333^†^
2: Patient centred goals are displayed appropriately in the patient's room [Model of care recommendation]	1	7	12	.010	.732^†^	.214	.266
3: Patient is able to identify primary rehabilitation goals consistent with documented goals from interdisciplinary family meeting [Model of care recommendation]	4	6	5	1.0	.076	.569	.262
4: Up-to-date treatment plan is in place [26]	5	6	17	1.0	.135	.606	.118
5: Documented goals guide and inform therapy and treatment [43]	2	8	14	.007	.775^‡^	.141	.330^†^
6: Staff are able to identify primary rehabilitation goals consistent with documented goals from interdisciplinary family meeting [Model of care recommendation]	7	8	13	1.0	.258	.142	.365^†^
**Continuity of care**							
1: Engagement with visitors is evident throughout a clear welcoming process [Model of care recommendation]	1	6	13	*	*	*	*
2: A patient centred care approach is used on the unit throughout the entire patient journey [10,25,27,40,42,43,44]	2	8	18	.015	.730^‡^	.577	.175
3: Continuity of care is in place for nursing [Model of care recommendation]	0	8	14	.0001^§^	1.0^‡^	.141	.330^†^
4: Continuity of care is in place for allied health [Model of care recommendation]	1	8	16	*	*	.295	.258
5: Continuity of care is in place for medicine [Model of care recommendation]	1	8	20	*	*	*	*
6: Patient/ family/informal caregivers are involved in the care planning meeting on the unit. [10,27,42,43]	1	7	18	.005	.854^‡^	1.0	.121
7: Escalation of patient issues or concerns has been documented appropriately [Model of care recommendation]	1	6	13	*	*	*	*
8: Engagement with family/informal caregiver is evident throughout every stage of recovery. [medical notes] [11,27]	5	8	20	.200	.480^†^	*	*
9: Engagement with family/informal caregiver is evident throughout every stage of recovery. [family report] [11, 27]	2	5	10	.021	.732^‡^	.559	.236
**Discharge planning**							
1: Interdisciplinary and patient (and family) directed discharge plan development is in place [25,40,43,44]	5	6	7	1.0	.174	.165	.370^†^
2: Training of family/ informal caregivers occurs prior to discharge: including safe use of equipment and management of the patient to ensure patient & caregiver safety in the home environment [medical notes] [25, 43] (a minimum of 4 weeks)	1	2	0	*	*	*	*
3: Assessment of discharge destination environment and available support occurs prior to discharge [25, 43] (a minimum of 4 weeks)	0	5	4	.167	1.0^‡^	.455	.430^†^
4: All required equipment and adaptations are provided prior to discharge [25]	*	1	0	*	*	1.0	1.0^‡^
5: Training of family/ informal caregivers occurs prior to discharge: including safe use of equipment and management of the patient to ensure patient & caregiver safety in the home environment [family report] [25, 43] (a minimum of 4 weeks prior)	1	1	1	*	*	*	*
6: Educating patients and family/informal caregivers about relevant formal and informal resources and how to access these resources including voluntary services and groups occurs prior to discharge [26, 43]	0	1	1	1.0	.333^†^	1.0	.577^‡^
7: Minimum of two weeks (before discharge) are spent in the transitional living space [26]	3	3	1	*	*	1.0	.250
**Equipment use**							
1: Instructions for the patient’s individualised equipment use is in place [43]	7	8	14	1.0	.258	1.0	.156
2: If prescribed, ceiling track hoist is used for every transfer within the past week [Model of care recommendation]	1	4	3	.333	.632^‡^	1.0	.378^†^
3: All staff are aware of the patient’s individualised equipment needs [medical notes] [Model of care recommendation]	7	6	20	1.0	.277	.259	.331^†^
4: All staff are aware of the patient’s individualised equipment needs [ask staff] [Model of care recommendation]	7	8	20	*	*	*	*
**Patient/family education [11]**							
1: Ward orientation	3	7	16	.119	.516^‡^	1.0	.020
2: Diet/nutrition	2	0	1	.487	.337^†^	1.0	.141
3: Psychosocial changes after ABI	1	7	15	.010	.750^‡^	1.0	.101
4: Wounds/lines/drains/airways	0	2	2	1.0	.316^†^	.547	.234
5: Tracheostomy care	*	1	1	*	*	*	*
6: Goal setting and rehabilitation importance	3	8	16	.026	.674^‡^	.532	.229
7: Discharge planning	1	7	11	.010	.750^‡^	.201	.287
8: Patient/family centred care	2	8	17	.007	.775^‡^	.567	.184
9: Diagnosis/illness/injury	1	6	16	.041	.630^‡^	.616	.108
10: Medical procedures/treatments	1	1	7	1.0	1.0^‡^	.364	.243
11: Safety	1	8	10	.001	.882^‡^	.026	.459^†^
12: Activity/mobility	0	7	8	.001	.882^‡^	.043	.417^†^
13: Self-care ADLs within the ward	1	7	6	.010	.750^‡^	.030	.500^‡^
14: Pain management	0	3	1	.200	.480^†^	.091	.395^†^
15: Medication management	0	0	5	*	*	.280	.309^†^
16: Equipment use	1	8	9	.001	.882^‡^	.115	.410^†^
**Goal setting**							
1: Patient has commenced goals setting within 48 hours of admission [11]	8	8	14	*	*	.277	.287
2: Goal-based planning meeting has taken place [11, 26] (within 2 weeks of admission)	0	8	13	.0001^§^	1.0^‡^	.142	.365^†^
**Medical management**							
1: Family / caregivers trained in the medical management plans for paretic upper limbs during transfers, hypersensitivity, and neurogenic pain are in place [26]	1	4	2	.143	.730^‡^	*	*
2: Benzodiazepines and Neuroleptic antipsychotics use minimised [10]	4	6	14	.608	.189	1.0	.030
3: Medication for Executive Dysfunction follows recommended guidelines [26]	*	*	0	*	*	*	*
4: Medication for management of memory is in place [26]	*	*	0	*	*	*	*
5: Stimulants are prescribed for management of memory as appropriate [26]	*	*	0	*	*	*	*
6: Medication for Arousal and Attention is prescribed appropriately [26,40]	2	2	0	*	*	*	*
7: Pain management plans are regularly reviewed [26]	7	8	19	*	*	*	*
8: Medical management plans for paretic upper limbs during transfers, hypersensitivity, and neurogenic pain are in place [26]	2	4	6	.429	.471^†^	1.0	.239
9: Appropriate medication management of agitation/ aggression is in place [26,40]	3	3	4	*	*	.500	.378^†^
10: Appropriate medication management of spasticity is in place [10,40,43]	0	3	5	.100	1.0^‡^	*	*
11: Appropriate medication management of mood and seizures is in place [26]	1	3	18	.400	.612^‡^	*	*
**Medical records**							
1: All invasive procedures are documented in accordance with hospital policies [Hospital policy]	1	8	20	.001	.882^‡^	*	*
2: Records only contain accurate statements of fact or clinical judgement [41]	7	8	20	1.0	.258	*	*
3: Records only contain abbreviations which are accepted and commonly known [Hospital policy]	4	8	20	.077	.577^‡^	*	*
**Minimally conscious care**							
1: Patients in a Coma, Vegetative and Minimal Conscious State are screened using a consistent assessment of recovery [40]	*	1	1	*	*	*	*
2: The Coma Recovery Scale -Revised has been administered consistently [40]	*	1	1	*	*^0^	*	*
3: Multisensory stimulation for patient in a coma or vegetative state is not carried out as an intervention [40]	*	1	1	*	*	*	*
**Safety**							
1: During the past week, the patient was sitting out of bed on morning of observation before 8am [Model of care recommendation]	0	4	13	.467	.408^†^	.359	.265
2: Safe diet strategies are in place [Model of care recommendation]	7	8	19	1.0	.258	*	*
3: Safe diet strategies are followed [Model of care recommendation]	7	8	19	1.0	.258	*	*
4: During the past week, the patient was sitting out of bed for all meals [Model of care recommendation]	2	4	14	1.0	.333^†^	.576	.167
5: All patients are screened for their fall risk as soon as practicable after admission [hospital policy]	*	8	20	*	*	*	*
6: All patients are screened for their pressure injury/sore risk as soon as practicable after admission [hospital policy]	*	8	20	*	*	*	*
7: All staff working with patients can identify safe transferring strategies [43]	8	8	20	*	*	*	*
**Personal care regime**							
1: Maximum privacy during use of the toilet at all times [Model of care recommendation]	*	4	10	*	*	*	*
2: All patients will have showers at a regular time each day consistent with their pre-injury showering time [Model of care recommendation] [medical notes]	0	4	10	.200	1.0^‡^	*	*
3: Patient personal care regimes are documented to ensure consistency between staff & with the aim of maximising independence [Model of care recommendation]	6	6	15	*	*	1.0	.000
4: All patients have a personalised toileting regime in place, at a regular time each day [Model of care recommendation]	1	0	2	1.0	.189	1.0	.222
5: All patients will have showers at a regular time each day consistent with their pre-injury showering time [Model of care recommendation] [ask patient]	1	5	14	.103	.577^‡^	.557	.195
**Post traumatic amnesia management**							
1: The Westmead PTA Scale (WPTAS) is commenced within 24 hours of emerging from coma and used to assess all patients following closed TBI [45]	2	2	1	*	*	*	*
2: The Orientation Log (O-Log) is commenced within 24 hours of emerging from coma for all other neurological patients (open TBI, stroke, hypoxic brain injury) [45]	*	*	1	*	*	1.0	1.0^‡^
3: The WPTAS /O-Log is administered by a consistent member of appropriately trained staff. (Clinical guidelines) [45]	1	4	8	.333	.632^‡^	.516	.333^†^
4: The WPTAS/O-Log is administered at a consistent time each day [Model of care recommendation]	0	4	10	.067	1.0^‡^	1.0	.218
5: Patients in PTA receive goal-oriented and procedural therapy (no new learning) [45]	4	5	4	*	*	1.0	.333^†^
**Roles and responsibilities**							
1: Roles and responsibilities for the implementation of the patient’s care are in place for family/caregivers and have been discussed with family [Model of care recommendation]	0	5	8	.008	1.0^‡^	.261	.358^†^
2: Roles and responsibilities for the implementation of the patient’s care are followed by the family/informal caregivers [Model of care recommendation]	4	5	9	*	*	.542	.255
3: Patient and/or their families/ informal caregivers are involved in the provision of patient care [Model of care recommendation]	5	6	11	*	*	1.0	.171
4: Roles and responsibilities for the implementation of the patient’s care are in place for family/informal caregivers [Model of care recommendation]	0	7	12	.001	.882^‡^	.214	.266
5: Roles and responsibilities for the implementation of the patient’s care are followed by the family/informal caregivers [Model of care recommendation]	0	7	12	.0001^§^	1.0^‡^	.273	.303^†^
6: Patient and/or their families/ informal caregivers are involved in the provision of patient care as much as they wish [26]	5	8	19	.200	.480^†^	1.0	.122
**Therapy**							
1: All appropriate patients are screened by a speech and language therapist within 48 hours of admission [26]	7	8	18	*	*	.577	.175
2: Seating plans are communicated with the family/informal caregivers [Model of care recommendation]	1	4	5	*	*	*	*
3: A therapy timetable is in place for each patient [Model of care recommendation]	7	8	18	1.0	.258	1.0	.127
4: Therapy is provided in the appropriate context for the individual [Model of care recommendation]	1	8	20	.200	.667^‡^	*	*
5: Learning and memory aids are in place in patient's room [Model of care recommendation]	5	8	19	.200	.419^†^	1.0	.122
6: Management of motor function and control is in place and follows evidenced based guidelines [10,11,25,26]	0	7	14	.001	.882^‡^	1.0	.000
7: Therapy is provided in the appropriate context for the individual [26, 42]	1	8	20	.003	.861^‡^	*	*
8: Leisure and recreation activities are included in the patient's weekly program [26, 42]	4	2	10	.608	.258	.236	.254
9: Seating needs are assessed within the required timeframe [Model of care recommendation]	4	8	20	.077	.535^‡^	*	*
10: Seating plans are followed by all staff. [Model of care recommendation]	1	7	12	.010	.837^‡^	*	*
11: Patients with a visual impairment have been assessed as per guidelines [10,11,25,26,40,43,44]	0	4	6	.167	.632^‡^	1.0	.000
12: Patients received a minimum of 4 hours of therapy per day at least 5 days a week in the past week [Model of care recommendation]	0	2	3	.467	.378^†^	1.0	.098
13: There is documented evidence that patients have received therapy from at least 3 different professions during the past week [Model of care recommendation]	6	8	19	.467	.378^†^	1.0	.122
14: Effective treatment approaches for rehabilitation are in place and embedded in daily life activities [10]	4	7	10	.282	.405^†^	.190	.330^†^
15: Learning and memory aids are in place and documented [42, 45]	3	7	20	.070	.632^‡^	*	*
16: If ‘15’ Is Yes: Patient is trained in the use of one, single external aid to compensate for memory impairments [Model of care recommendation]	2	6	18	.103	.537^‡^	1.0	.150
17: Errorless learning approach / scripts are documented [Model of care recommendation]	0	2	8	.091	.632^‡^	1.0	.060
18: Interventions addressing poor executive functioning are in place [45]	1	1	0	.250	.655^‡^	.167	1.0^‡^
19: Repetition of computer based tasks are not carried out unless additional cognitive rehabilitation strategies are used [45]	3	2	7	*	*	*	*
20: Staff are aware of seating plan [Model of care recommendation]	4	7	19	.192	.461^†^	*	*
**Ward round**							
1: Documented evidence of that the weekly ward round includes ANUM and the patient nurse in addition to RMO/Resident and rehabilitation physician [41]	2	0	0	.467	.378^†^	*	*
2: Documented evidence of the weekly ward round records nursing dependency data [Model of care recommendation]	*	*	1	*	*	1.0	.122
3: Documented evidence that ward rounds are taken to each patient (inclusive of therapy spaces) [Model of care recommendation]	0	8	20	.0001^§^	1.0^‡^	*	*
4: Documented evidence that weekly ward rounds include discussion of: basic care needs, specialised nursing needs, dependency on nursing time for common tasks, and influences on dependency [41]	*	*	1	*	*	1.0	.122

* = Unable to compute as some items responses are ‘not applicable’

† = medium effect size[41]

‡ = large effect size[41]

§ statistically significant at the Bonferroni adjusted p-value 0.000217

## Discussion

Our sustained fortnightly audit and feedback program led to a significant increase in adherence to clinical practice guideline recommendations. Following the three-month cessation period during which no audit and feedback was provided, adherence to guideline recommendations decreased (but remained above the organization’s benchmark of ≥75% adherence). The positive results of our study contrast to other audit and feedback studies conducted in rehabilitation [15,16,17]. Our program had strong support from senior management and the organization, as well as external funding. This external context supported higher frequency audit and feedback cycles, and our feedback was grounded in social cognitive modelling. The adherence improvements following intervention were likely due to a combination of the following attributes of our program: a) high level of managerial support, b) feedback delivered using a non-aversive and clinician-led approach, c) high frequency of audit and feedback cycles, d) 12-month duration of the program, and e) shared goal of working towards a target of ≥75% adherence. By describing these attributes, future studies can build on our program’s success.

We do acknowledge that when the audit and feedback program was ceased, adherence rates decreased, although they did not return to baseline levels. This decrease was not unexpected, and while we did not investigate the reasons why, we anticipate that the loss of accountability (knowledge that auditing was not occurring) as well as no longer having formal opportunities to reflect on practice gaps contributed to the lower rates of adherence. Interestingly, there were some audit indicators that increased in adherence after the program was ceased which suggests that comprehensive processes developed and established during the study period carried over beyond the period of audit and feedback.

Our results support many findings from audit and feedback studies conducted outside of rehabilitation. Indicators that had high adherence at baseline in our study were also less likely to improve with regular audit and feedback [12, 13, 30, 31]- the benefits of audit and feedback programs are likely greatest when baseline performance is low. The use of positive support while delivering feedback (i.e. employing a ‘no blame’ ethos and highlighting discipline ‘achievements’) is also consistent with other studies [18, 32, 33] which suggest that when feedback which is perceived as supportive rather than punitive, it is more likely to positively influence clinician behavior. Finally, our study provided feedback in both written and verbal formats by a respected internal senior member of staff. These characteristics are described in systematic reviews as effective strategies to increase audit and feedback effectiveness [12, 13]. Future studies testing audit and feedback interventions should continue to investigate models of providing feedback.

**Fig 3:**
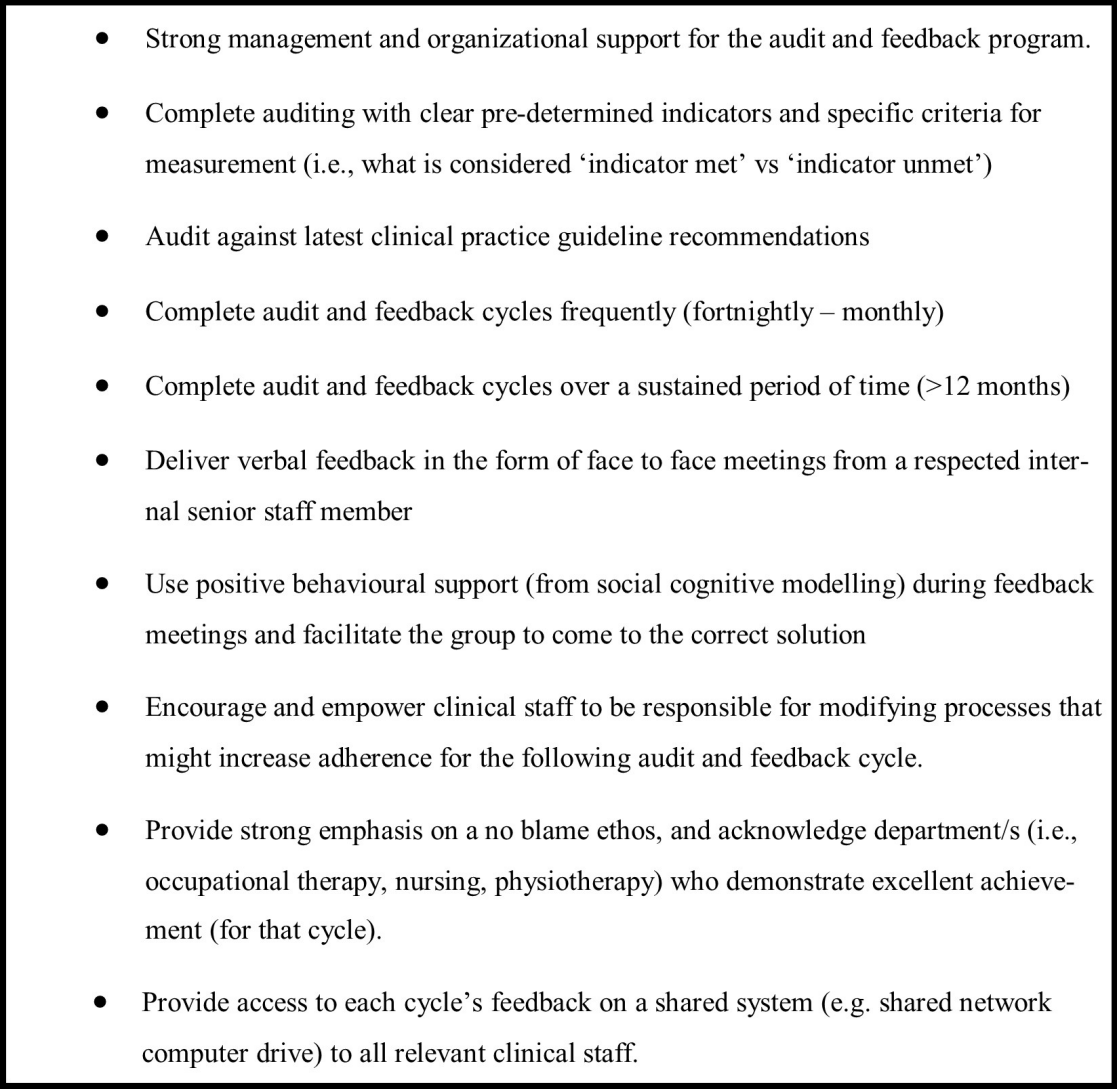
Factors that contribute to the success of the audit and feedback program as indicated by the present study.

Setting targets (or goals) has been proposed as increasing the effectiveness of feedback, however, this remains uncertain [34, 35]. In contrast to Garner and colleagues[36], our results suggest that setting goals and developing action plans during feedback sessions was an effective strategy. With positive support, the facilitator guided clinician discussions towards solutions and encouraged the clinicians to create changes that may lead to increased guideline adherence for the following fortnight. The use of a cognitive model, in combination with high frequency (i.e., fortnightly) and solution-focused feedback is a novel addition to the evaluative studies in this field and supported in theory by the work of Hysong [13] and Ivers [12, 31]. Fig 3 outlines these potential factors which may have contributed to the success of the audit and feedback program.

Organizational expectation of clinician participation was likely to contribute to the high level of staff engagement achieved in the present study. Current behavior change models focus predominantly on individual level or local change characteristics (i.e. the Behaviour Change Wheel [37] and Theoretical Domains Framework [38]). Research around behavior change interventions have explored staff motivation for and perceptions of audit and feedback on an individual level [18]. Less discussed is how organizational expectations drive behavior change in clinicians. The revisited Promoting Action on Research Implementation (PARiHS) framework aptly encompasses the construct of environment and context; separating out micro (local) and meso (organizational) from macro (political, policy) levels [39]. In this framework, organizational systems and culture are a key consideration for behavior change. Given the organizational expectation of staff involvement in our current study, as well as the intervention frequency (i.e. fortnightly) and paid staff time release for feedback, the strong contribution of organization and culture to our positive findings cannot be overlooked.

### Study limitations

Like all pragmatic studies in the clinical setting, our study is not without limitations. Not all staff attended each fortnight’s feedback session. While this reflects the practical reality of a ward environment and the shiftwork nature of hospital staffing, it did mean that not all clinicians received regular feedback. This study sought to investigate the effectiveness of a sustained program, and so this was an accepted limitation within the design of the study. We also acknowledge that the use of only one site may limit the generalizability of the results. The use of only one site also limits our ability to predict whether scaling up will achieve similar rates of adoption and delivery across multiple organizations. Furthermore, contextual factors may have positively affected the uptake at our study site (since it was newly established with newly employed staff) which may not directly translate to other sites. Our program also sought to improve adherence to n = 114 indicators of best-practice rehabilitation. While effective at the single site, scaling up our complex audit and feedback intervention may not be straightforward and future programs may choose a smaller number of indicators to implement. Finally, this was a funded study, so sustainable infrastructure needs to be established to enable scaling up. We recommend that future studies include a controlled comparison, consider using both publically and privately funded rehabilitation hospitals, and include a cost/benefit analysis alongside any evaluation of efficacy.

## Conclusion

Our study demonstrated that a frequent and sustained audit and feedback program is an effective knowledge translation intervention to increase adherence to brain injury rehabilitation guidelines. Findings also highlighted that some guideline recommendation indicators that are less likely to change with audit and feedback, suggesting that alternative knowledge translation strategies may be more appropriate to achieve behavior change for these items. Our program has the potential to inform both local and larger initiatives to improve the quality of rehabilitation received, and more significantly beyond rehabilitation, in the field of implementation science and the knowledge base underpinning audit and feedback.

## Supporting information

S1 Table Standards for Reporting Implementation Studies: the StaRI checklist for completion. (DOCX)

S2 Table Proportion (%) (95% CI) of clinical practice guideline indicator adherence (n = 114) across measurement points. (DOCX)
