# Supplementary material for: Using audit and feedback to increase clinician adherence to clinical practice guidelines in brain injury rehabilitation: A before and after study
Source: PLoS One. 2019 Mar 13;14(3):e0213525. doi: 10.1371/journal.pone.0213525 (PMC6415863; doi:10.1371/journal.pone.0213525)
Supplement: S2 Table — (DOCX) [file pone.0213525.s002.docx]

**S2 Appendix. Proportion (%) (95% CI) of clinical practice guideline indicator adherence (n=114) across measurement points**

| Clinical practice guideline indicator | Percent (%) of clinical practice adherence obtained at three time points | | | | | | Difference between groups (mean, 95% CI) | | |
| --- | --- | --- | --- | --- | --- | --- | --- | --- | --- |
|  | 0-2 months (baseline) |  | 13-15 months (post intervention) |  | 18-19 months (follow-up) |  | 13-15 months minus 0-2 months |  | 18-19 months minus 13-15 months |
| Behavioural support plans, 1 | 100  (100 to 100) |  | 100  (100 to 100) |  | 50  (12 to 88) |  | 0  * |  | -50 (-175 to 75) |
| Behavioural support plans, 2 | 38 (-6 to 81) |  | 100 (100 to 100) |  | 95 (84.5 to 106) |  | 62.5 (19.2 to 105.8) |  | -5 (-21.4 to 11.4) |
| Behavioural support plans, 3 | 40 (-28.0 to 108.0) |  | 100 (100 to 100) |  | 100 (100 to 100) |  | 60 (-19.9 to 139.9) |  | 0 * |
| Behavioural support plans, 4 | 50 (-42 to 142) |  | 100 (100 to 100) |  | 100 (100 to 100) |  | 50 (-37.8 to 137.8) |  | 0 * |
| Behavioural support plans, 5 | 100 (100 to 100) |  | 100 (100 to 100) |  | 100 (100 to 100) |  | 0 * |  | 0 * |
| Behavioural support plans, 6 | 67 (-77 to 210) |  | 33 (-110 to 117) |  | 83 (59 to 108) |  | -33 (-164.2 to 97.5) |  | 50 (-9.1 to 109.1) |
| Care Plan, 1 | 60 (-8 to 128) |  | 100 (100 to 100) |  | 67 (35 to 98) |  | 40 (-25.7 to 105.7) |  | 33.3 (-87.4 to 20.7) |
| Care Plan, 2 | 14 (-21 to 49) |  | 88 (58 to 117) |  | 60 (36 to 84) |  | 73.2 (32.4 to 114) |  | -27.5 (-67.7 to 12.7) |
| Care Plan, 3 | 80 (24 to 136) |  | 86 (51 to 121) |  | 63 (19 to 106) |  | 5.7 (-47.4 to 58.8) |  | -23.2 (-74.5 to 28) |
| Care Plan, 4 | 63 (19 to 106) |  | 75 (36 to 114) |  | 85 (68 to 102) |  | 12.5 (-40.2 to 65.2) |  | 10 (-23.9 to 43.9) |
| Care Plan, 5 | 25 (-14 to 64) |  | 100 (100 to 100) |  | 70 (48 to 92) |  | 75 (36.3 to 113.7) |  | -30 (-64.6 to 4.6) |
| Care Plan, 6 | 88 (58 to 117) |  | 100 (100 to 100) |  | 65 (42 to 88) |  | 12.5 (-14.3 to 39.3) |  | -35 (-71.0 to 1.0) |
| Continuity of care, 1 | 100 (100 to 100) |  | 100 (100 to 100) |  | 100 (100 to 100) |  | 0 * |  | 0 * |
| Continuity of care, 2 | 33 (-21 to 88) |  | 100 (100 to 100) |  | 90 (76 to 104) |  | 66.7 (12.5 to 120.9) |  | -10 (-32.6 to 12.6) |
| Continuity of care, 3 | 70 (48 to 92) |  | 100 (100 to 100) |  | 70 (48 to 92) |  | 30 (8 to 52) |  | -30 (-64.6 to 4.6) |
| Continuity of care, 4 | 100 (100 to 100) |  | 100 (100 to 100) |  | 80 (61 to 99) |  | 0 * |  | -20 (-50.2 to 10.2) |
| Continuity of care, 5 | 100 (100 to 100) |  | 100 (100 to 100) |  | 100 (100 to 100) |  | 0 * |  | 0 * |
| Continuity of care, 6 | 17 (-26 to 60) |  | 100 (100 to 100) |  | 95 (84 to 106) |  | 83.3 (40.5 to 126.2) |  | -5.3 (-23.4 to 12.9) |
| Continuity of care, 7 | 100 (100 to 100) |  | 100 (100 to 100) |  | 100 (100 to 100) |  | 0 * |  | 0 * |
| Continuity of care, 8 | 63 (19 to 106) |  | 100 (100 to 100) |  | 100 (100 to 100) |  | 37.5 (-5.8 to 80.8) |  | 0 * |
| Continuity of care, 9 | 25 (-14 to 64) |  | 100 (100 to 100) |  | 83 (59 to 108) |  | 75 (36.3 to 113.7) |  | -16.7 (-54.5 to 21.2) |
| Discharge planning, 1 | 71 (26 to 117) |  | 86 (51 to 121) |  | 47 (18 to 75) |  | 14.3 (-36.5 to 65.1) |  | -39 (-84.8 to 6.7) |
| Discharge planning, 2 | 100 (100 to 100) |  | 100 (100 to 100) |  | * |  | 0 * |  | * |
| Discharge planning, 3 | 0 * |  | 100 (100 to 100) |  | 67 (12 to 121) |  | 100 (100 to 100) |  | -33 (-86.1 to 19.4) |
| Discharge planning, 4 | * |  | 100 (100 to 100) |  | 0 * |  | * |  | 100 * |
| Discharge planning, 5 | 100 (100 to 100) |  | 100 (100 to 100) |  | 100 (100 to 100) |  | 0 * |  | 0 * |
| Discharge planning, 6 | 0 * |  | 33 (-110 to 177) |  | 100 (100 to 100) |  | 33.3 * |  | 66.7 (-220 to 353.5) |
| Discharge planning, 7 | * |  | 75 (-5 to 155) |  | 100 (100 to 100) |  | * |  | 25 (-152.9 to 202.9) |
| Equipment use, 1 | 88 (58 to 117) |  | 100 (100 to 100) |  | 93 (79 to 108) |  | 12.5 (-14.3 to 39.3) |  | -6.7 (-25.9 to 12.5) |
| Equipment use, 2 | 50 (-585 to 685) |  | 100 (100 to 100) |  | 75 (-5 to 155) |  | 50 (-35 to 135) |  | -25 (-86.2 to 36.2) |
| Equipment use, 3 | 100 (100 to 100) |  | 86 (51 to 121) |  | 100 (100 to 100) |  | -14.3 (-45.4 to 16.8) |  | 14.3 (-2.5 to 31.0) |
| Equipment use, 4 | 100 (100 to 100) |  | 100 (100 to 100) |  | 100 (100 to 100) |  | 0 * |  | 0 * |
| Family Education, 1 | 38 (-6 to 81) |  | 88 (58 to 117) |  | 89 (73 to 105) |  | 50 (1.9 to 98.1) |  | 1.4 (-27.8 to 30.5) |
| Family Education, 2 | 25 (-14 to 64) |  | 0 * |  | 7 (-8 to 23) |  | -25 (-71.3 to 21.3) |  | 7.1 (-18.5 to 32.8) |
| Family Education, 3 | 13 (-17 to 42) |  | 88 (58 to 117) |  | 79 (59 to 99) |  | 75 (37.1 to 112.9) |  | -8.6 (-43.4 to 26.3) |
| Family Education, 4 | 0 * |  | 40 (-28 to 108) |  | 18 (-9 to 45) |  | 40 (-126.6 to 206.6) |  | -21.8 (-73.9 to 30.3) |
| Family Education, 5 | * |  | 100 (100 to 100) |  | 100 (100 to 100) |  | * |  | 0 * |
| Family Education, 6 | 38 (-6 to 81) |  | 100 (100 to 100) |  | 84 (66 to 102) |  | 62.5 (19.2 to 105.8) |  | -15.8 (-43.4 to 11.8) |
| Family Education, 7 | 13 (-17 to 42) |  | 88 (58 to 117) |  | 58 (33 to 82) |  | 75 (37.1 to 112.9) |  | -29.6 (-70.3 to 11.1) |
| Family Education, 8 | 25 (- 14 to 64) |  | 100 (100 to 100) |  | 89 (74 to 105) |  | 75 (36.3 to 113.7) |  | -10.5 (-33.7 to 12.7) |
| Family Education, 9 | 13 (-17 to 42) |  | 75 (36 to 114) |  | 84 (66 to 102) |  | 62.5 (18 to 107) |  | 9.2 (-25.6 to 44) |
| Family Education, 10 | 13 (-17 to 42) |  | 13 (-17 to 42) |  | 37 (13 to 61) |  | 0 (-37.9 to 37.9) |  | 24.3 (-15.6 to 64.3) |
| Family Education, 11 | 13 (-17 to 42) |  | 100 (100 to 100) |  | 53 (28 to 77) |  | 87.5 (57.9 to 117.1) |  | -47.4 (-72.1 to -22.6) |
| Family Education, 12 | 0 * |  | 88 (58 to 117) |  | 42 (18 to 67) |  | 87.5 (57.9 to 117.1) |  | -45.4 (-81.2 to -9.6) |
| Family Education, 13 | 13 (-17 to 42) |  | 88 (58 to 117) |  | 33 (9 to 57) |  | 75 (37.1 to 112.9) |  | -54.2 (-89.7 to -18.6) |
| Family Education, 14 | 0 * |  | 38 (-6 to 81) |  | 6 (-7 to 20) |  | 38 (-1.7 to 76.7) |  | -31.3 (-63.47 to 0.9) |
| Family Education, 15 | 0 * |  | 0 * |  | 26 (5 to 48) |  | 0 * |  | 26.3 (-7.0 to 59.6) |
| Family Education, 16 | 13 (-17 to 42) |  | 100 (100 to 100) |  | 64 (36 to 93) |  | 87.5 (57.9 to 117.1) |  | -35.7 (-72.8 to 1.3) |
| Goal setting, 1 | 100 (100 to 100) |  | 100 (100 to 100) |  | 78 (57 to 99) |  | 0 * |  | -22.2 (-53.8 to 9.4) |
| Goal setting, 2 | 0 * |  | 100 (100 to 100) |  | 65 (42 to 88) |  | 100 (100 to 100) |  | -35 (-71 to 1) |
| Medical management, 1 | 33 (-110 to 177) |  | 100 (100 to 100) |  | 100 (100 to 100) |  | 66.7 (-5.0 to 138.4) |  | 0 * |
| Medical management, 2 | 57 (8 to 107) |  | 75 (36 to 114) |  | 78 (57 to 99) |  | 17.9 (-37.7 to 73.5) |  | 2.8 (-35.7 to 41.2) |
| Medical management, 3 | 0 * |  | * |  | * |  | * |  | * |
| Medical management, 4 | 0 * |  | * |  | * |  | * |  | * |
| Medical management, 5 | 0 * |  | * |  | * |  | * |  | * |
| Medical management, 6 | 33 (-21 to 88) |  | * |  | * |  | * |  | * |
| Medical management, 7 | 100 (100 to 100) |  | 100 (100 to 100) |  | 100 (100 to 100) |  | 0 * |  | 0 * |
| Medical management, 8 | 67 (-77 to 210) |  | 100 (100 to 100) |  | 86 (51 to 121) |  | 33.3 (-38.4 to 105) |  | -14.3 (-58 to 29.5) |
| Medical management, 9 | 100 (100 to 100) |  | 100 (100 to 100) |  | 67 (12 to 121) |  | 0 * |  | -33.3 (-106.3 to 39.6) |
| Medical management, 10 | 0 * |  | 100 (100 to 100) |  | 100 (100 to 100) |  | 100 (100 to 100) |  | 0 * |
| Medical management, 11 | 50 (585 to 685) |  | 100 (100 to 100) |  | 100 (100 to 100) |  | 50 (-68.6 to 168.6) |  | 0 * |
| Medical records, 1 | 13 (-17 to 42) |  | 100 (100 to 100) |  | 100 (100 to 100) |  | 87.5 (57.9 to 117.1) |  | 0 * |
| Medical records, 2 | 88 (58 to 117) |  | 100 (100 to 100) |  | 100 (100 to 100) |  | 12.5 (-14.3 to 39.3) |  | 0 * |
| Medical records, 3 | 50 (5 to 95) |  | 100 (100 to 100) |  | 100 (100 to 100) |  | 50 (5.3 to 94.7) |  | 0 * |
| Minimally conscious care, 1 | * |  | 100 (100 to 100) |  | 100 (100 to 100) |  | * |  | 0 * |
| Minimally conscious care, 2 | * |  | 100 (100 to 100) |  | 100 (100 to 100) |  | * |  | 0 * |
| Minimally conscious care, 3 | * |  | 100 (100 to 100) |  | 100 (100 to 100) |  | * |  | 0 * |
| Safety, 1 | 0 * |  | 50 (5 to 95) |  | 76 (54 to 99) |  | 50 (-41.2 to 141.2) |  | 26.5 (-15.1 to 68.1) |
| Safety, 2 | 88 (58 to 117) |  | 100 (100 to 100) |  | 100 (100 to 100) |  | 12.5 (-14.3 to 39.3) |  | 0 * |
| Safety, 3 | 88 (58 to 117) |  | 100 (100 to 100) |  | 100 (100 to 100) |  | 12.5 (-14.3 to 39.3) |  | 0 * |
| Safety, 4 | 100 (100 to 100) |  | 67 (12 to 121) |  | 82 (62 to 103) |  | -33 (-127.5 to 60.8) |  | 15.7 (-26.3 to 57.7) |
| Safety, 5 | * |  | 100 (100 to 100) |  | 100 (100 to 100) |  | * |  | 0 * |
| Safety, 6 | * |  | 100 (100 to 100) |  | 100 (100 to 100) |  | * |  | 0 * |
| Safety, 7 | 100 (100 to 100) |  | 100 (100 to 100) |  | 100 (100 to 100) |  | 0 * |  | 0 * |
| Personal care regime, 1 | * |  | 100 (100 to 100) |  | 100 (100 to 100) |  | * |  | 0 * |
| Personal care regime, 2 | 0 * |  | 100 (100 to 100) |  | 100 (100 to 100) |  | 100 (100 to 100) |  | 0 * |
| Personal care regime, 3 | * |  | 75 (36 to 114) |  | 75 (54 to 96) |  | * |  | 0 (-38.6 to 38.6) |
| Personal care regime, 4 | 14 (-21 to 49) |  | 0 * |  | 22 (-12 to 56) |  | -14.3 (-80.6 to 52.1) |  | 22.2 (-51.3 to 95.7) |
| Personal care regime, 5 | 14 (-21 to 49) |  | 71 (26 to 117) |  | 88 (69 to 106) |  | 57.1 (6.0 to 108.3) |  | 16.1 (-20.6 to 52.7) |
| PTA Management, 1 | 100 (100 to 100) |  | 100 (100 to 100) |  | 100 (100 to 100) |  | 0 * |  | 0 * |
| PTA Management, 2 | * |  | 0 * |  | 100 (100 to 100) |  | * |  | 100 * |
| PTA Management, 3 | 50 (-585 to 685) |  | 100 (100 to 100) |  | 67 (35 to 98) |  | 50 (-35.0 to 135.0) |  | -33 (-87.4 to 20.7) |
| PTA Management, 4 | 0 * |  | 100 (100 to 100) |  | 83 (59 to 108) |  | 100 (100 to 100) |  | -16.7 (-59.4 to 26.1) |
| PTA Management, 5 | 100 (100 to 100) |  | 100 (100 to 100) |  | 80 (24 to 136) |  | 0 * |  | -20 (-66.1 to 26.1) |
| Roles and responsibilities, 1 | 0 * |  | 100 (100 to 100) |  | 67 (35 to 98) |  | 100 (100 to 100) |  | -33.3 (-81.2 to 14.5) |
| Roles and responsibilities, 2 | 100 (100 to 100) |  | 100 (100 to 100) |  | 82 (55 to 109) |  | 0 * |  | -18.2 (-57.7 to 21.4) |
| Roles and responsibilities, 3 | 100 (100 to 100) |  | 100 (100 to 100) |  | 92 (73 to 110) |  | 0 * |  | -8.3 (-33.7 to 17) |
| Roles and responsibilities, 4 | 0 * |  | 88 (58 to 117) |  | 60 (36 to 84) |  | 87.5 (57.9 to 117.1) |  | -27.5 (-67.7 to 12.7) |
| Roles and responsibilities, 5 | 0 * |  | 100 (100 to 100) |  | 75 (51 to 99) |  | 100 (100 to 100) |  | -25 (-60.6 to 10.6) |
| Roles and responsibilities, 6 | 63 (19 to 106) |  | 100 (100 to 100) |  | 95 (85 to 105) |  | 37.5 (-1.7 to 76.7) |  | -5 (-21.4 to 11.4) |
| Therapy, 1 | 100 (100 to 100) |  | 100 (100 to 100) |  | 90 (76 to 104) |  | 0 * |  | -10 (-32.6 to 12.6) |
| Therapy, 2 | 100 (100 to 100) |  | 100 (100 to 100) |  | 100 (100 to 100) |  | 0 * |  | 0 * |
| Therapy, 3 | 88 (58 to 117) |  | 100 (100 to 100) |  | 95 (84 to 106) |  | 12.5 (-17.1 to 342.1) |  | -5.3 (-22.2 to 11.6 |
| Therapy, 4 | 50 (-585 to 685) |  | 100 (100 to 100) |  | 100 (100 to 100) |  | 50 (4.4 to 95.6) |  | 0 * |
| Therapy, 5 | 71 (26 to 117) |  | 100 (100 to 100) |  | 95 (85 to 105) |  | 28.6 (-16.6 to 73.7) |  | -5 (-21.4 to 11.4) |
| Therapy, 6 | 0 * |  | 88 (58 to 117) |  | 88 (69 to 106) |  | 87.5 (57.9 to 117.1) |  | 0 (-31 to 31) |
| Therapy, 7 | 17 (-26 to 60) |  | 100 (100 to 100) |  | 100 (100 to 100) |  | 83.3 (40.5 to 126.2) |  | 0 * |
| Therapy, 8 | 50 (5 to 95) |  | 25 (-14 to 64) |  | 53 (28 to 77) |  | -25 (-78.6 to 28.6) |  | 27.6 (-15.7 to 71.0) |
| Therapy, 9 | 57 (8 to 108) |  | 100 (100 to 100) |  | 100 (100 to 100) |  | 42.9 (-6.6 to 92.3) |  | 0 * |
| Therapy, 10 | 20 (-36 to 76) |  | 100 (100 to 100) |  | 100 (100 to 100) |  | 80 (24.5 to 135.5) |  | 0 * |
| Therapy, 11 | 0 * |  | 67 (12 to 121) |  | 67 (28 to 105) |  | 66.7 (12.5 to 120.9) |  | 0 (-57.7 to 57.7) |
| Therapy, 12 | 0 * |  | 25 (-14 to 64) |  | 17 (-20 to 36) |  | 25 (-13.7 to 63.7) |  | -8.3 (-44.1 to 27.5) |
| Therapy, 13 | 75 (36 to 114) |  | 100 (100 to 100) |  | 95 (85 to 105) |  | 25 (-13.7 to 63.7) |  | -5 (-21.4 to 11.4) |
| Therapy, 14 | 50 (5 to 95) |  | 88 (58 to 117) |  | 53 (28 to 77) |  | 37.5 (-11.8 to 86.8) |  | -34.9 (-76.0 to 6.3) |
| Therapy, 15 | 43 (-7 to 92) |  | 100 (100 to 100) |  | 100 (100 to 100) |  | 57.1 (7.7 to 106.6) |  | 0 * |
| Therapy, 16 | 33 (-21 to 88) |  | 86 (51 to 121) |  | 95 (84 to 106) |  | 52.4 (-2.3 to 107) |  | 9 (-16 to 34) |
| Therapy, 17 | 0 * |  | 50 (-42 to 142) |  | 57 (27 to 87) |  | 50 (-41.9 to 141.9) |  | 7.1 (-56.1 to 70.4) |
| Therapy, 18 | 14 (-21 to 49) |  | 100 (100 to 100) |  | 0 * |  | 85.7 (-13.2 to 184.6) |  | -100 * |
| Therapy, 19 | 100 (100 to 100) |  | 100 (100 to 100) |  | 100 (100 to 100) |  | 0 * |  | 0 * |
| Therapy, 20 | 67 (12 to 121) |  | 100 (100 to 100) |  | 100 (100 to 100) |  | 33.3 (-20.9 to 87.5) |  | 0 * |
| Ward round, 1 | 25 (-14 to 64) |  | 0 * |  | 0 * |  | -25 (-63.7 to 13.7) |  | 0 * |
| Ward round, 2 | 0 * |  | 0 * |  | 5 (-5 to 15) |  | * |  | 5 (-11.4 to 21.4) |
| Ward round, 3 | 0 * |  | 100 (100 to 100) |  | 100 (100 to 100) |  | 100 (100 to 100) |  | 0 * |
| Ward round, 4 | 0 * |  | 0 * |  | 5 (-5 to 15) |  | * |  | 5 (-11.4 to 21.4) |

* = Unable to compute as some items responses are ‘not applicable’ or contain a score of ‘0’, CI = Confidence Interval
